# The genome sequence of *Rhynchonycteris naso, Peters, 1867 *(Chiroptera, Emballonuridae, Rhynchonycteris)

**DOI:** 10.12688/wellcomeopenres.19959.1

**Published:** 2024-07-10

**Authors:** Ine Alvarez van Tussenbroek, Mirjam Knörnschild, Martina Nagy, Brian P. O'Toole, Giulio Formenti, Philip Philge, Ning Zhang, Linelle Abueg, Nadolina Brajuka, Erich Jarvis, Thomas L. Volkert, Jonathan L. Gray, Myrtani Pieri, Meike Mai, Emma C. Teeling, Sonja C. Vernes, The Bat Biology Foundation, The Bat1K Consortium

**Affiliations:** 1 School of Biology, University of St Andrews, St Andrews, Scotland, UK; 2 Neurogenetics of Vocal Communication Group, Max Planck Institute for Psycholinguistics, Nijmegen, Gelderland, The Netherlands; 3 Institute of Biology, Leiden University, 2300 RA Leiden, PO Box 9505, The Netherlands; 4 Museum für Naturkunde, Leibniz-Institute for Evolution and Biodiversity Science, Berlin, Germany; 5 Institute for Biology, Humboldt-Universität zu Berlin, Berlin, Germany; 6 Smithsonian Tropical Research Institute, Balboa Ancon, Panama City, Panama; 7 Paratus Sciences, New York, USA; 8 Vertebrate Genome Laboratory, The Rockefeller University, New York, New York, USA; 9 Excelra, Hyderabad, India; 10 Whitehead Institute of Biomedical Research, Cambridge, Massachusetts, USA; 11 Department of Life Sciences, University of Nicosia, Nicosia, Nicosia, Cyprus; 12 School of Biology and Environmental Science,, University College Dublin, Dublin, Ireland; 13 Wellcome Genome Campus, Wellcome Sanger Institute, Cambridgeshire, England, CB10 1SA, UK

**Keywords:** Rhynchonycteris naso, genome sequence, chromosomal, Bat1K

## Abstract

We present a reference genome assembly from an individual male *Rhynchonycteris naso* (Chordata; Mammalia; Chiroptera; Emballonuridae). The genome sequence is 2.46 Gb in span. The majority of the assembly is scaffolded into 22 chromosomal pseudomolecules, with the Y sex chromosome assembled.

## Species taxonomy

Eukaryota; Metazoa; Chordata; Craniata; Vertebrata; Euteleostomi; Mammalia; Eutheria; Laurasiatheria; Chiroptera; Yangochiroptera; Emballonuroidea; Emballonuridae; Emballonurinae; *Rhynchonycteris*; *Rhynchonycteris naso ^1– 4^*.

## Introduction

Emballonurid bats are aerial insectivores. They are found in Africa and Indo-Malayan, Australian, Neotropical, and Holarctic regions. Although typically found in tropical forest regions, a few species have been found in semiarid and desert regions ^5^.

**Figure 1.**
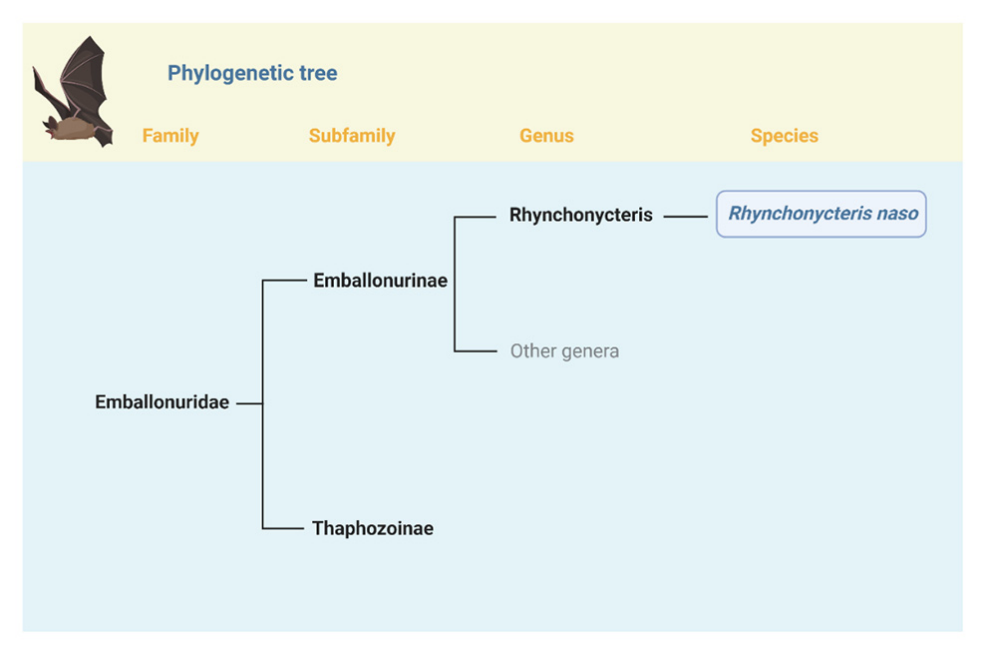
Position of *Rhynchonycteris naso* in the phylogeny of Emballonuridae. The bat *Rhynchonycteris naso* is the only species currently recognized in the genus *Rhynchonycteris ^13^. Rhynchonycteris naso* belongs to the Subfamily Emballonurinae, which currently includes 14 genera and 55 species ^6^.

The Emballonuridae family comprises two subfamilies: Taphozoinae and Emballonurinae. Emballonurinae consists of 14 genera and 55 species ^6^. The genus *Rhynchonycteris* is within Emballonurinae and comprises a sole species: *Rhynchonycteris naso* ( *Rhynchonycteris* is one of four monotypic genera in Emballonuridae) ( Figure 1).

**Figure 2.**
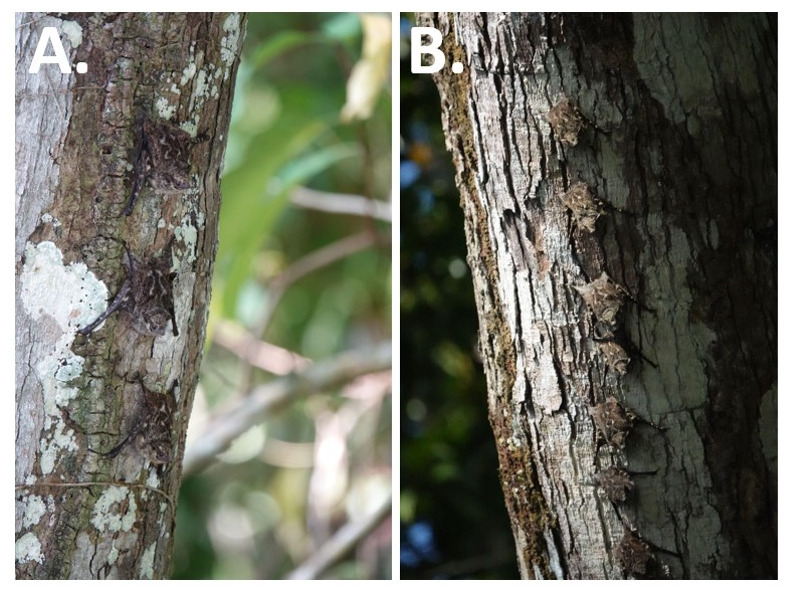
Proboscis bats, *Rhynchonycteris naso* Individuals of *R. naso*. ( **A**– **B**) These bats roost in a line formation often on trees and near the water. They sometimes look like tree bark or lichen due to their grey and brown marbled coat and light stripes on their backs [Photos taken near a river close to Gamboa, Panama by Ine Alvarez van Tussenbroek].

*Rhynchonycteris naso,* the proboscis bat, has been found in tropical regions in middle and south America from the south of Mexico to the north of Bolivia and center of Brazil ^5^. Proboscis bats are found up to 1500 meters elevation, generally at less than 500 meters elevation, often in lowland tropical forest, close to water bodies ^5^. They roost in an exposed manner on tree trunks or man-made structures in the vicinity of water ^7^. Their grey and brown marbled coat makes them well camouflaged, they often look like tree bark (see Figure 2) or may be perceived as swaying leaves since they may form a single line along the tree’s length ( Figure 2A–B) and can be observed rocking back and forth ^5, 8^. They live in stable multi-male-multi-female groups of usually <40 individuals ^7^. Male mating strategies are based on both direct female-defense and male territoriality ^9, 10^. A substantial proportion of males are philopatric ^9^.

*Rhynchonycteris naso* has been commonly referred to as long-nosed and or sharp-nosed bat in reference to the nose protruding from the rest of the face. They are small bats 36–48 mm body size with added ~11–17 mm of tail length, the forearm length is ~36–40 mm and they weigh around 3–6 g ^5^. Although the family Emballonuridae is sometimes referred to as the “sac-winged bats”, *R. naso* lacks wing sacs ^7^. *R. naso* is classified in the IUCN Red List as a species of Least Concern.

*Rhynchonycteris naso* hunts small dipterans (such as mosquitoes, flies and caddisflies) ^5, 7^. The echolocation calls of *R. naso* are CF-FM with the CF component around 100 kHz during search flight ^11^. The echolocation call frequency is lowered to 67 kHz during prey capture to maintain the peripheral acoustic view ^12^. This strategy is different from the other members of the Emballonuridae family which use a constant frequency throughout the whole pursuit sequence.

## Genome sequence report

The genome was sequenced from a single male *R. naso* collected on March 9th 2019, from a tree near the river in Gamboa, Panama (GPS coordinates: 9.1135734185584, -79.82011865195433). A total of 42x-fold coverage in Pacific Biosciences Hi-Fi long reads (contig N50 20 Mb) was generated after removal of all reads shorter than 10kb. Primary assembly contigs were scaffolded with chromosome conformation (Hi-C) data, which was also used to attain chromosome-level phasing ^14^. The final assembly has a total length of 2.455 Gb in 40 sequence scaffolds with a contig N50 of 86 Mbp scaffold N50 of 286 Mbp ( Table 1). The assembly has a BUSCO ^15^ completeness of 95.3% using the laurasiatheria reference set. Chromosomal pseudomolecules in the genome assembly of *Rhynchonycteris naso* are shown in Table 2.

**Table 1.** Genome data for *Rhynchonycteris naso*.

*Project accession data*	
Assembly identifier	GCA_031021685.1
Species	*Rhynchonycteris naso*
Specimen	rhynas1
NCBI taxonomy ID	249017
BioProject	PRJNA1076651, PRJNA1076652 Bat1K: Accession: PRJNA489245; ID: 489245
BioSample ID	SAMN39947078
Isolate information	Male [heart]
*Raw data accessions*	
Pacific Biosciences SEQUEL II	SRS20636215
Hi-C Illumina	SRS20636215
Genome assembly	
Assembly accession	GCA_037038545.1
Assembly of alternative accession	GCA_037038555.1
Span (Mb)	2455
Number of contigs	108
Contig N50 length (Mb)	86.3
Number of scaffolds	40
Scaffold N50 length (Mb)	287
Longest scaffold (Mb)	372

* BUSCO scores based on the laurasiatheria_odb10 BUSCO set using v5.0.0. C= complete [S= single copy, D=duplicated], F=fragmented, M=missing, n=number of orthologues in comparison. * *Rhynchonycteris naso* BUSCO scores based on laurasiatheria_odb10 BUSCO set v5.3.2.

**Table 2.** Chromosomal pseudomolecules in the genome assembly of *Rhynchonycteris naso*. ENA accession Chromosome Size (Mb) GC%. The chromosome number of *Rhynchonycteris naso* is 2n=22.

ENA Accession	Chromosome	Size (Mb)	GC%
SUPER_1	1	372.38	0.4189
SUPER_2	2	317.77	0.3968
SUPER_3	3	310.926	0.4063
SUPER_4	4	286.99	0.3953
SUPER_5	5	261.06	0.4168
SUPER_6	6	209.12	0.4066
SUPER_7	7	170.32	0.4314
SUPER_8	8	151.66	0.3915
SUPER_X	X	142.67	0.3867
SUPER_9	9	132.37	0.4223
SUPER_10	10	78.41	0.4207
SUPER_Y	Y	19.81	0.3984

## Methods

The *R. naso* specimen was a male individual collected during a field expedition in Gamboa, Panama. *Rhynconycteris naso* was first identified by the roost location (a group of *R. naso* bats were hanging from a tree trunk in a line formation close to shallow waters). Furthermore, the shape of the face with a protruding nose, the gray-brown fur and the two light colored lines on the back of these bats determined the identification of this species as described previously (e.g. 9, 16. After going on a boat by the river close to Gamboa, a roost was spotted on a tree near the water near a location previously investigated by locals under the supervision of the expert fieldworkers Mirjam Knörnschild and Martina Nagy. The bat was caught using a hand net and after confirmation of the sex it was placed in a fabric bag and taken to the laboratories at the Smithsonian Institute in Gamboa for tissue harvesting. Capture and sampling were done under the project proposal 2019-0301-2022 approved by the Smithsonian Tropical Research Institute and the STRI Animal Care and Use Committee (ACUC) and collection and export was conducted under the collecting field number issued by UNARGEN SC/A-3-19. All work was conducted with approval by the Panamanian Ministry of Environment (Mi Ambiente). Tissues were removed from the subject individual immediately following euthanasia and were flash-frozen in liquid nitrogen and stored in a freezer at -80°C until shipping on dry ice, maintaining the cold chain.

All efforts were made to minimize any suffering of the animal. The animal was subjected to minimal handling after capture, and it was held in a clean cloth bag after capture as per best practices for field containment of bats ^17^. After species identification, the individual was euthanized humanely by experienced researchers while monitoring and prioritizing the reduction of stress and suffering of the animal. The animal was euthanized by overdose of isoflurane inhalation (Formula CHF _2_OCClHCF _3_, CAS number 26675-46-7; Manufacturer Piramal Critical Care, Supplier US Pharmacy Systems, Product code 5034-1FL-SOL-ORA). Euthanasia by isoflurane inhalation is a humane approved method that rapidly causes unconsciousness and eventually death upon inhalation. Bats euthanized by this method are rendered unconscious within seconds due to their high respiration rate, and death occurs within a minute or two with no significant suffering by the animal. The animal was tested for absence of breathing and reflexes. After breathing stops, isoflurane exposure was extended for one more minute. Confirmation of death was done immediately by decapitation. Tissue samples were dissected and immediately snap frozen using liquid nitrogen. A total of 21 samples were collected including brain, blood, liver, spleen, heart, lung, testes, muscle and kidney. All data were recorded and reported in accordance with the ARRIVE guidelines – see data availability section and Table 1.

DNA was extracted using Nanobind extraction from muscle tissue following the Circulomics Nanobind HMW DNA Extraction Protocol. Pacific Biosciences HiFi libraries were constructed according to the manufacturer's instructions. Hi-C data was generated using the Arima Hi-C+ High Coverage kit from the same muscle tissue sample. Sequencing was performed by the Genomic Operations DNA Pipelines at Paratus Sciences on Pacific Biosciences Sequel IIe (HiFi reads) and Illumina NextSeq 2000 (Hi-C) instruments.

**Figure 3.**
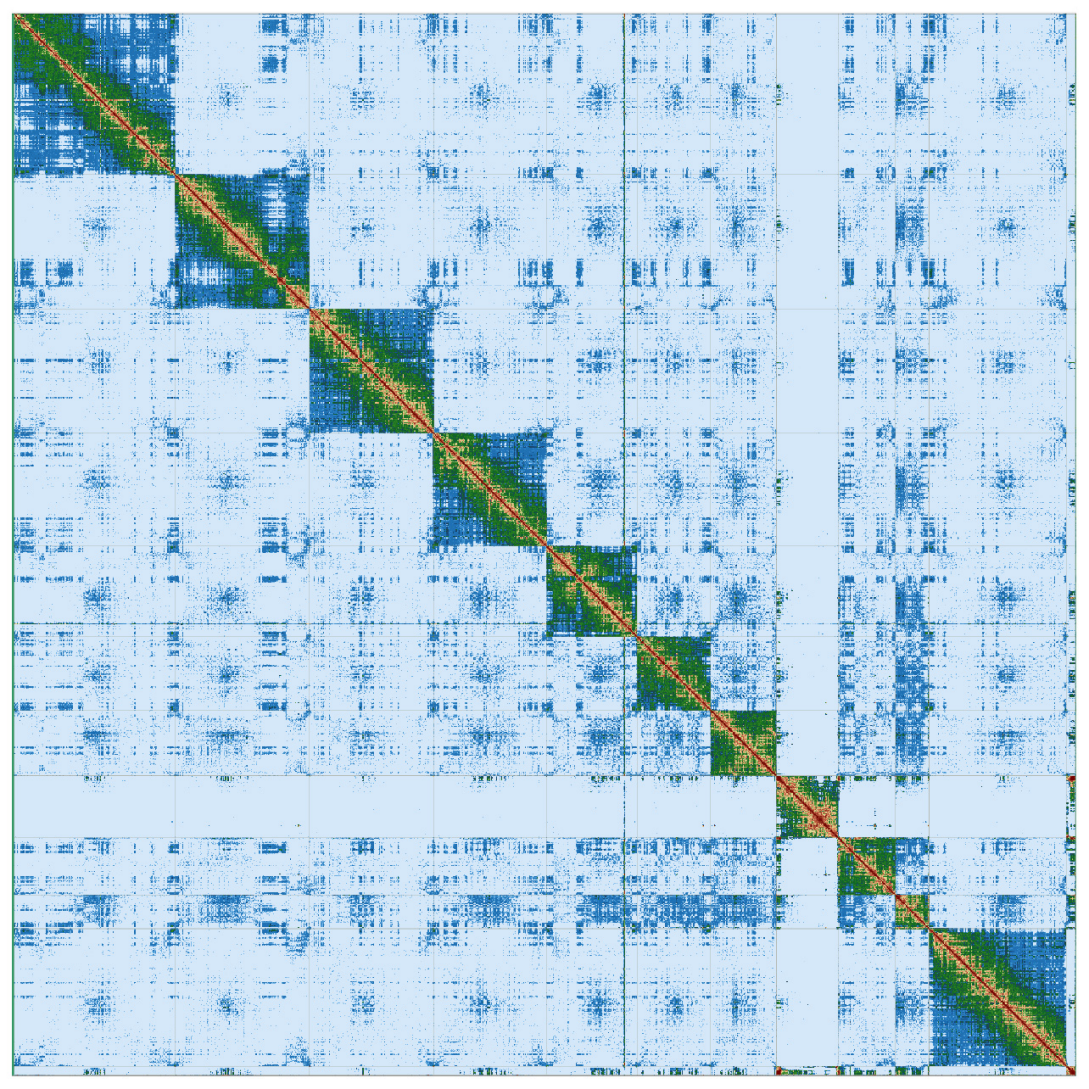
Hi-C Contact Map of the Rhynchonycteris naso haplotype 1 assembly with 11 scaffolds, visualized using PretextView. Scaffolds below 10 Mb were removed for creating the Hi-C Contact Map.

**Figure 4.**
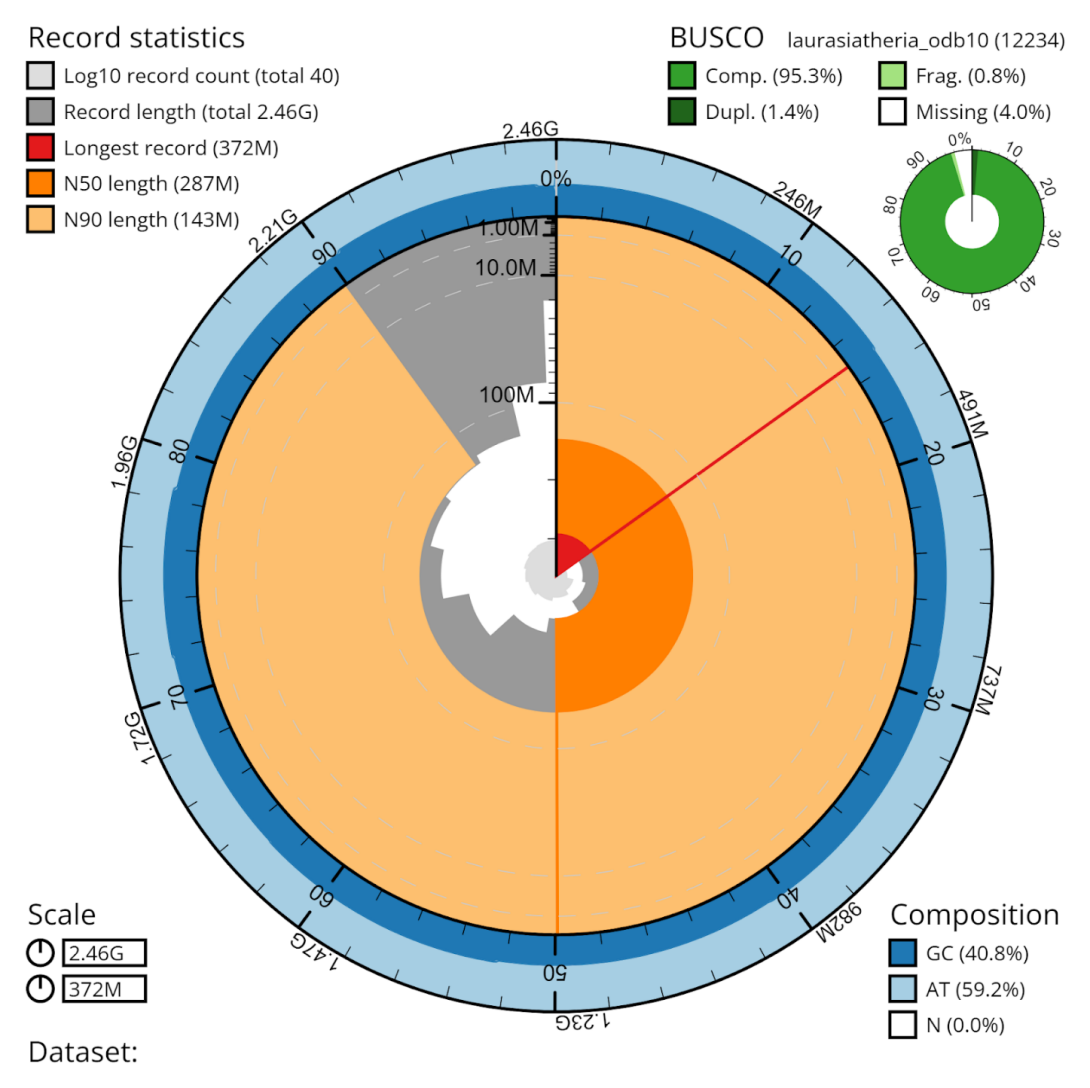
Genome assembly metrics generated using blobtoolkit for the *Rhynchonycteris naso* genome assembly. The larger snail plot depicts scaffold statistics including N50 length (bright orange) and base composition (blue). The smaller plot shows BUSCO completeness in green.

Assembly was carried out following the Vertebrate Genome Project Galaxy pipeline v2.0 ^18^. A brief synopsis of the method is as follows: Genome size was estimated using GenomeScope2 ^19^. Hifiasm with Hi-C phasing was used for genome assembly (Cheng, Haoyu *et al.* 2021). The quality of the assembly was evaluated using Merqury ^20^ and BUSCO ^21^. Scaffolding with Hi-C data (Rao, Huntley *et al.* 2014) was carried out with YaHS (Zhou, McCarthy *et al.* 2023). PretextView was implemented to generate a Hi-C contact map ( Figure 3). Figure 4– Figure 6 were generated using BlobToolKit ^22^. All bioinformatics software utilised for the *R. naso* analysis are depicted in Table 3.

**Figure 5.**
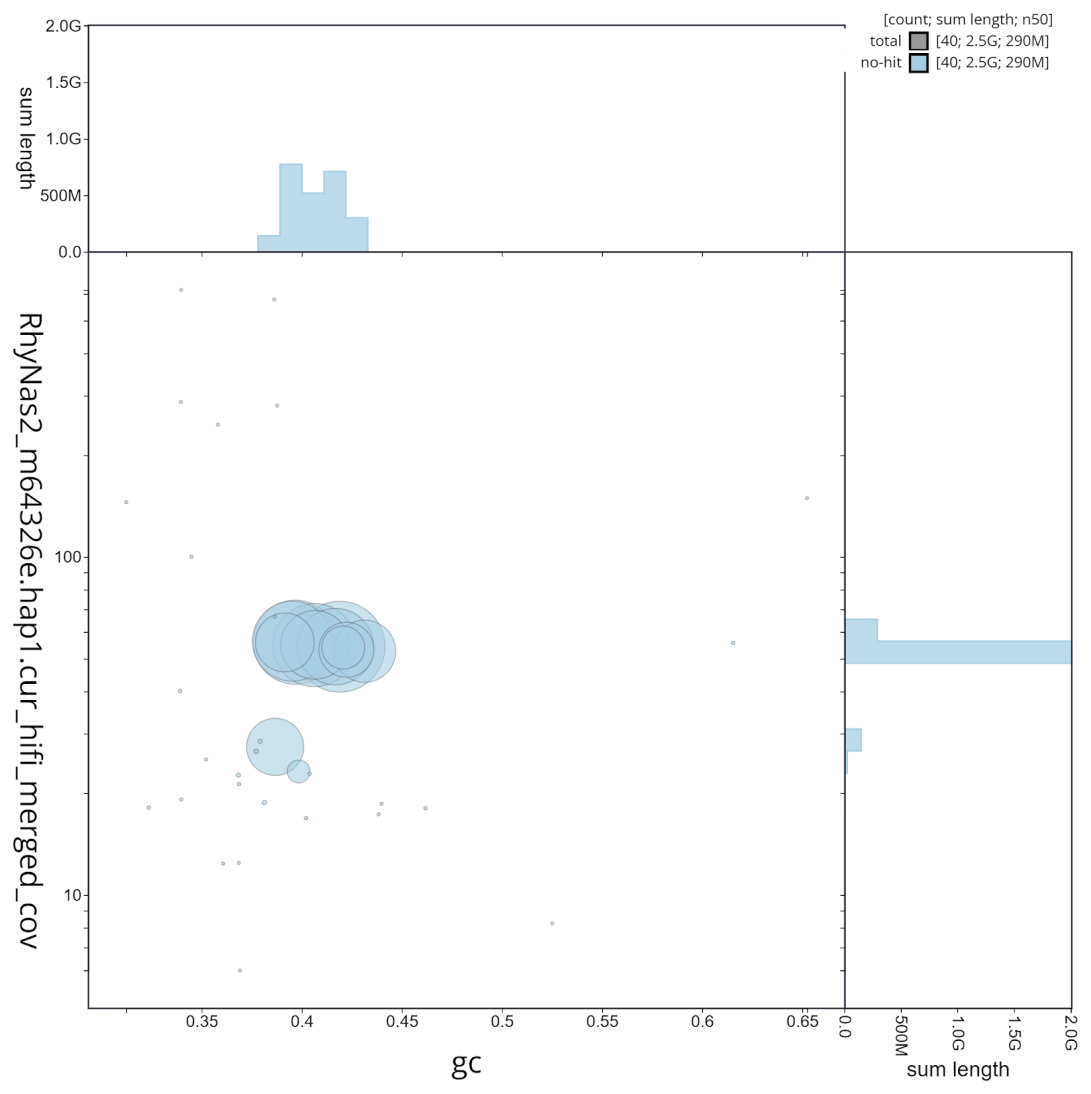
GC coverage plot generated for the *Rhynchonycteris naso* assembly using blobtoolkit. Individual chromosomes and scaffolds are represented by each circle. The circles are sized in proportion to chromosome/scaffold length. Histograms show the sum length of chromosome/scaffold size along each axis. Color of circles indicate taxonomic hits of each Phylum represented in the assembly.

**Figure 6.**
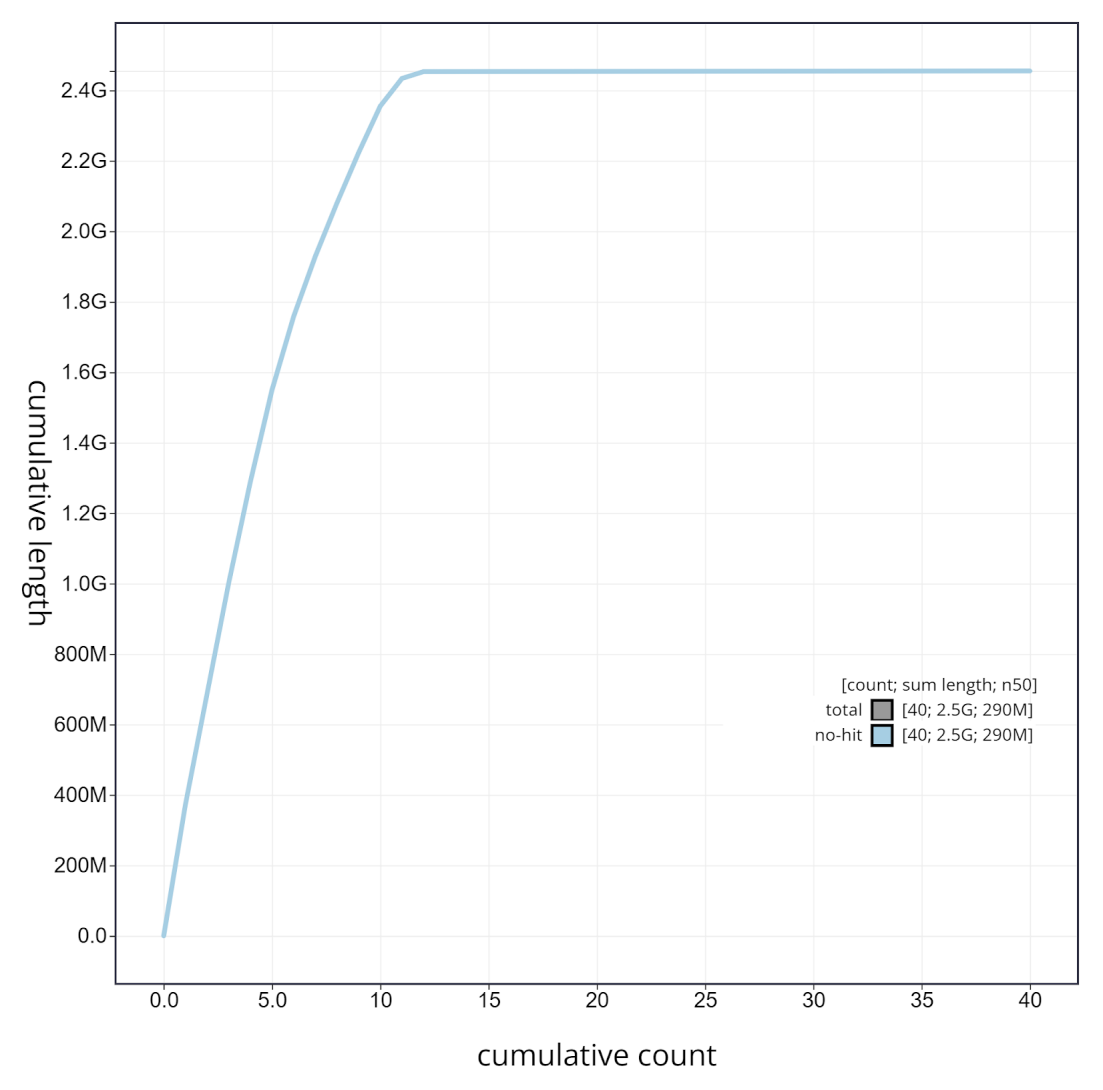
Cumulative sequence plot generated for the *Rhynchonycteris naso* assembly using blobtoolkit. The grey line shows the cumulative length for all chromosomes/scaffolds in the assembly. Colored lines represent Phylum represented in the assembly.

**Table 3.** Software tools used.

Software tool	Version	Source
bamUtil	1.0.15	https://genome.sph.umich.edu/wiki/BamUtil:_bam2FastQ
MultiQC	1.13	https://github.com/ewels/MultiQC
Genomescope	2.0	https://github.com/tbenavi1/genomescope2.0
hifiasm	0.19.3	https://github.com/chhylp123/hifiasm
purge_dups	1.2.6	https://github.com/dfguan/purge_dups
BUSCO	5.3.2	https://busco.ezlab.org/
Merqury	1.3	https://github.com/marbl/merqury
Assembly-stats	17.02	https://github.com/rjchallis/assembly-stats
Arima-HiC Mapping Pipeline	-	https://github.com/ArimaGenomics/mapping_pipeline
YaHS	1.1	https://github.com/c-zhou/yahs
HiGlass	1.11.7	https://github.com/higlass/higlass
samtools	1.9	https://www.htslib.org/
PretextView	-	https://github.com/sanger-tol/PretextView/tree/master
BUSCO	5.7.0	https://busco.ezlab.org/
BlobToolKit	4.3.5	https://github.com/blobtoolkit/blobtoolkit
pbmm2	1.13.1	https://github.com/PacificBiosciences/pbmm2
Blast	2.15.0+	https://blast.ncbi.nlm.nih.gov/Blast.cgi

## Data Availability

The *Rhynchonycteris naso* genome sequencing initiative is part of the Bat1K genome sequencing project. The genome assembly is released openly for reuse. Underlying data may be available for non-commercial research purposes upon request. Please email info@batbio.org for more information. The genome assembly for *Rhynchonycteris naso* (proboscis bat) can be found in the European Nucleotide Archive and NCBI. The assembly accession number at NCBI is GCA_031021685.1, and more details can be accessed through this link: https://www.ncbi.nlm.nih.gov/datasets/genome/GCA_031021685.1/. NCBI BioProject: Rhynchonycteris naso isolate: mRhyNas1 *(proboscis bat)*. Accession number: PRJNA945050, http://identifiers.org/ncbiprotein:PRJNA945050 ^23^ under the Bat1K BioProject PRJNA489245. The genome assembly can be found in the European Nucleotide Archive: Rhynchonycteris naso (proboscis bat). Accession number GCA_037038555, https://www.ebi.ac.uk/ena/browser/view/GCA_037038555.1 ^24^. All raw sequence data and the assembly have been deposited in the ENA (PRJNA1076651, PRJNA1076652) and NCBI (raw data). Data accession identifiers are SAMN39947078. Data accession identifiers are reported in Table 1.
