# Supplementary material for: The genome sequence of Rhynchonycteris naso, Peters, 1867 (Chiroptera, Emballonuridae, Rhynchonycteris)
Source: Wellcome Open Res. 2024 Jul 10;9:361. [Version 1] doi: 10.12688/wellcomeopenres.19959.1 (PMC11375410; doi:10.12688/wellcomeopenres.19959.1)
Supplement: Peer review history

# Reviewer report: Reviewer response for version 1

**DOI:** 10.21956/wellcomeopenres.22103.r93044

**Published:** 2024-09-04

**Authors:** Luciano Chaves Franco Filho

**Affiliations:** 1 Instituto Evandro Chagas, Ananindeua, Brazil

The Data Note presents a high-quality reference genome assembly for the proboscis bat, *Rhynchonycteris naso*, from the family Emballonuridae. The genome, covering around 2.46 Gb, is organized into 22 chromosomal pseudomolecules, including the Y chromosome. The assembly achieved a contig N50 of 86 Mb and a scaffold N50 of 287 Mb, demonstrating the completeness and accuracy of the genome sequence. This work contributes to the Bat1K project, aiming to sequence the genomes of all bat species, and provides valuable insights into the genetic makeup of *R. naso*, an insectivorous bat species found in tropical regions of Central and South America.

Are sufficient details of methods and materials provided to allow replication by others?

Yes

Is the rationale for creating the dataset(s) clearly described?

Yes

Are the datasets clearly presented in a useable and accessible format?

Yes

Are the protocols appropriate and is the work technically sound?

Yes

Reviewer Expertise:

Next Generation Sequencing (NGS); Molecular biology; biodiversity and microbiome diversity

I confirm that I have read this submission and believe that I have an appropriate level of expertise to confirm that it is of an acceptable scientific standard.

# Reviewer report: Reviewer response for version 1

**DOI:** 10.21956/wellcomeopenres.22103.r95139

**Published:** 2024-09-04

**Authors:** Wenhua Yu

**Affiliations:** 1 Guangzhou University, Guangzhou, Guangdong, China

Authors presented a exciting reference genome assembly from an individual male *Rhynchonycteris naso. *No doubt it will benefit the future genomic analyses on Emballonuridae and other.

**However, I have 2 minor comments for its improvement:**

 1) The use of *Rhynchonycteris naso *Peters, 1867 is not correct. I think it should be *Rhynchonycteris naso *(Wied-Neuwied, 1820). Pls check "Mammal species of the World" for the further details. 

 2) "Rhynchonycteris" in the Fig. 1 should be italic (genera name).

Are sufficient details of methods and materials provided to allow replication by others?

Yes

Is the rationale for creating the dataset(s) clearly described?

Yes

Are the datasets clearly presented in a useable and accessible format?

Yes

Are the protocols appropriate and is the work technically sound?

Yes

Reviewer Expertise:

Comparative genomics, Taxonomy, Evolutionary biology, Diversification, Bats

I confirm that I have read this submission and believe that I have an appropriate level of expertise to confirm that it is of an acceptable scientific standard.

# Reviewer report: Reviewer response for version 1

**DOI:** 10.21956/wellcomeopenres.22103.r95133

**Published:** 2024-09-02

**Authors:** Xiuguang Mao

**Affiliations:** 1 School of Ecological and Environmental Sciences, East China Normal University, Shanghai, China

This manuscript presents a high-quality chromosome-scale assembly for *Rhynchonycteris naso* and also provides detailed characteristics about this species in the Introduction section.

**I have two minor comments:**

 1) In Figure 1, ‘Rhynchonycteris’ should be italic.

 2) “The final assembly has a total length of 2.455 Gb in 40 sequence scaffolds with a contig N50 of 86 Mbp scaffold N50 of 286 Mbp (Table 1)”. But in Table 1, the scaffold N50 length is 287 Mb.

Are sufficient details of methods and materials provided to allow replication by others?

Yes

Is the rationale for creating the dataset(s) clearly described?

Yes

Are the datasets clearly presented in a useable and accessible format?

Yes

Are the protocols appropriate and is the work technically sound?

Yes

Reviewer Expertise:

Comparative genomics, genome assembly, evolutionary biology, speciation, bats

I confirm that I have read this submission and believe that I have an appropriate level of expertise to confirm that it is of an acceptable scientific standard.
